# Dr. Matthew Bevier

**Published:** 1908-04

**Authors:** 


Dr. Matthew Bevier, of Niles, N. Y., died at his home in that village, January 27, 1908, aged 87 years. He graduated at the University of Buffalo in 1848, and for more than 40 years practised his profession at Owasco, N. Y.

